# The Change Club intervention: 2-year impacts from a cluster-randomized trial in rural communities in New York and Texas

**DOI:** 10.3389/fpubh.2026.1793324

**Published:** 2026-07-02

**Authors:** Rebecca A. Seguin-Fowler, Karla L. Hanson, Galen D. Eldridge, Grace A. Marshall, Jay E. Maddock, Sara C. Folta, Deyaun L. Villarreal, Meredith L. Graham

**Affiliations:** 1 Institute for Advancing Health Through Agriculture (IHA), Texas A&M AgriLife Research, College Station, TX, United States; 2 Department of Nutrition, College of Agriculture and Life Sciences, Texas A&M University, College Station, TX, United States; 3 Department of Public and Ecosystem Health, Cornell University, Ithaca, NY, United States; 4 Institute for Advancing Health Through Agriculture, Texas A&M AgriLife Research, Dallas, TX, United States; 5 School of Public Health, Texas A&M University, College Station, TX, United States; 6 Friedman School of Nutrition Science and Policy, Tufts University, Boston, MA, United States

**Keywords:** built environment, civic engagement, nutrition, physical activity, PSE, randomized trial, rural

## Abstract

**Introduction:**

Obesity and related chronic diseases in the U.S., particularly within rural communities and/or medically underserved areas, are a major societal and cost burden. Built environments with health-promoting attributes, which can be influenced through civic engagement, are a potential pathway to help address this substantial problem.

**Methods:**

We conducted a community-randomized delayed intervention trial in medically underserved rural communities (*n* = 12) in Texas and New York to evaluate the impacts of a civic engagement curriculum designed to create healthier physical activity and nutrition environments, called the Change Club, on individual measures of chronic disease risk and related behaviors and on collective and environmental outcomes 2 years after baseline. Residents within each community were recruited and enrolled to help lead community change activities; they are referred to as Change Club Members.

**Results:**

Using an intent-to-treat framework, no net effects were observed when comparing year-2 individual and collective outcomes for the intervention arm relative to the control arm, adjusting for baseline outcome values. Exploratory analyses compared attendees (61%) to non-attendees (39%) in the intervention group and found attendees had higher year-2 values than non-attendees (with baseline adjustment) on the primary outcome and two other individual measures: Life’s Simple 7 composite cardiovascular risk score (+0.93, *p* = 0.006), World Cancer Research Fund/American Institute for Cancer Research composite cancer risk score (+0.54, *p* = 0.008), and healthy eating motivation (+0.27, *p* = 0.028).

**Discussion:**

This is among the first studies to evaluate how a civic engagement curriculum targeting built environment change may impact the health of residents who actively attend the Change Club meetings. While net impacts on the whole sample were not observed, exploratory analysis indicated that the approach holds promise when participants attend.

**Clinical trial registration:**

http://www.clinicaltrials.gov, identifier (NCT05002660).

## 1 Introduction

Nearly three-quarters of U.S. adults (73.1%) are overweight or obese (1). Excess body weight is associated with a wide range of health problems, including cardiovascular disease (CVD), some types of cancer, musculoskeletal problems, sleep issues, respiratory problems, and other conditions (2). Only about one-quarter of U.S. adults (24.2%) meet physical activity (PA) guidelines for both aerobic and muscle-strengthening activities (3). Inadequate PA is associated with premature death and increased chronic disease risk, including CVD and cancer (4). While consuming a healthy diet is associated with a lower risk of chronic diseases like diabetes, CVD, and some cancers (5), less than 13% of U.S. adults meet fruit and vegetable (FV) intake guidelines (6). Inadequate PA and FV intake are major contributors to healthcare costs; for example, it is estimated that inadequate aerobic PA costs the U.S. $192 billion per year in healthcare expenses, representing 12.6% of healthcare expenditures (7).

Rural populations in the U.S. have higher rates of CVD (8), obesity (9), physical inactivity (10), and poor diets (11) compared to urban populations. Rural areas also have higher poverty levels (12) and more limited access to healthcare, healthy food, PA facilities, and active transportation opportunities (13). Feasible and effective interventions are needed to improve healthy eating and PA opportunities in rural areas of the U.S.

The built environment is associated with both health (e.g., chronic diseases) and health behaviors (e.g., PA, healthy eating) (14). Changes in the built environment have shown potential to improve residents’ health (15–22). The importance of the built environment is recognized by the Centers for Disease Control and Prevention (23) and the World Health Organization (24). These organizations, among others, recommend improving the built environment to help people live healthier lives. Rural environments in the U.S. often present unique challenges to health-related behaviors, including geographic distances to access healthy food and PA opportunities, as well as active transport challenges; for example, in small rural communities, there is often poor infrastructure for walking or biking, with high speed limits for cars (25).

The social environment (e.g., social support, community norms, social cohesion, social networks) also influences PA and healthy eating behaviors (26–29). Rural residents often benefit from strong social networks and social cohesion, but these can also create barriers to healthy living, through community or social norms of unhealthy habits (e.g., age and gender norms for who uses public spaces for exercise, informal dissemination of safety concerns about exercise) (30, 31). Overlapping rural social networks with high social cohesion have strong potential to implement and evaluate civic engagement for policy, systems, and environmental (PSE) change interventions.

Civic engagement is defined as “individual and collective actions designed to identify and address issues of public concern” (32). In civic engagement for PSE change projects, groups of residents are guided through a process of assessing their community and developing and carrying out an action plan for PSE change. Civic engagement works at multiple levels of the socioecological model (33). At the individual level, civic engagement can positively impact cognitive influences; promote behavioral skills, including goal-setting and monitoring; and improve self-efficacy. At the group level, civic engagement can provide social support and improve collective efficacy, thereby impacting health behaviors (34–36). Civic engagement interventions, when aimed at PSE changes, can also improve the broader social and community environment by enhancing bonds of trust and identity within the group working together to improve PSE to make it easier for community residents to be physically active and eat healthfully. Behavior change may be further reinforced through reciprocal determinism, or a positive, reinforcing interaction among behavioral, cognitive, and environmental factors (37).

Several civic engagement for PSE change projects have led to meaningful PSE changes, including allocation of government funds for built environment improvements, sidewalk repair programs, addition of shade trees to encourage walking, and installation of pedestrian flashing light signals (38–46). In at least one study, civic engagement for PSE change projects has been associated with health improvements for participants (e.g., increased PA, improved aerobic fitness, blood pressure reduction) (38). Very few studies have evaluated individual-level health behavior changes or health outcomes in response to these projects, and most civic engagement PSE interventions were not evaluated using matched control communities (38, 47–50).

This study, which was delayed due to the Coronavirus-2019 (COVID-19) pandemic, estimated the impacts of the Change Club, a civic engagement PSE change program, on residents, using a cluster-randomized controlled trial (cRCT) design. The central hypothesis was that the Change Club would improve health behaviors and outcomes among engaged citizens and their social networks and that these changes could catalyze critical steps in the pathway to improving rural health equity by increasing opportunities for healthy eating and PA. The study had three objectives, two about individual-level outcomes and one focused on collective and environmental (cluster-level) outcomes. Three groups of residents were considered in the cRCT: Change Club Members (CCM) themselves and community residents, including a sub-sample of people in the CCMs’ social networks. This analysis reports only on the CCM sample.

*Aim 1*: To evaluate changes in *cardiovascular health* among CCM in intervention communities compared to comparable groups in control communities.

*Aim 2*: To evaluate changes in *individual health outcomes and behaviors, and adherence to cancer-related recommendations*, among CCM in intervention communities relative to comparable groups in control communities.

*Aim 3*: To evaluate changes in *civic engagement, social cohesion, and perceptions of the community environment* among CCM in intervention communities relative to comparable groups in control communities (cluster-level objective).

## 2 Methods

### 2.1 Trial design

This study used a cluster-randomized controlled design in which communities are the clusters, using a two-arm parallel design (51) with equal allocation. An intent-to-treat (ITT) framework was used because we aimed to estimate the effectiveness of Change Club in altering the health status and behaviors of participants under real-world conditions (52). A ‘per protocol’ framework was used secondarily to assess effectiveness among the sub-sample of participants who attended any Change Club meetings relative to all controls (53).

### 2.2 Participants

Eligibility criteria for communities were designated as at least partially rural (54) and as a medically underserved area and/or Health Professional Shortage Area (55). Furthermore, they had an interested and available Extension educator to lead the intervention. The Extension system is a nationwide educational network, based at land-grant universities, that shares research-based knowledge to help people with community issues, including agriculture, youth development, nutrition, and health. The study design required 12 communities (clusters) in six matched pairs. Twenty eligible communities were invited to recruit participants, as we anticipated that not every community would be able to successfully launch a Change Club due to enrollment challenges in rural areas. Seven communities were dropped because of recruitment difficulties, and an eighth community was discontinued because there were not at least three CCMs who could regularly attend synchronous meetings.

Study participants were recruited using letters and postcards to zip code mailing lists, social media posts, targeted digital advertising, emails sent to purchased contact lists, local media pieces, community events, and Extension educator outreach to groups and individuals (56). All CCM were 18 or older, lived in a participating community in New York or Texas, and provided electronic informed consent. They also scored “poor” or “intermediate” on at least one of the American Heart Association’s Life’s Simple 7 (LS7) composite score items and were willing to be randomized to either group. Due to high levels of fraud detected during the recruitment process [described elsewhere (57)], eligibility was confirmed at 1-year follow-up before the sample size was finalized (52). All participants (intervention and control) were compensated for data collection activities (51).

Data were collected electronically via the Qualtrics survey platform (Qualtrics, Provo, UT, USA). Responses to all survey questions were required, except potentially sensitive questions relating to income, food security, relationship status, or social determinants of health, which could be skipped. One 24-h dietary recall via the Automated Self-Administered 24-h Dietary Assessment Tool (ASA24), versions 2020 and 2024 (National Cancer Institute, Bethesda, MD), was completed. A new version of the ASA24 is released approximately every 2 years to make use of up-to-date information on available food products and their nutrients provided in an updated USDA Food and Nutrient Database for Dietary Surveys (FNDDS), and may also include minor changes to the user interface to improve functionality (58). Study participants also reported 7 days of pedometer/fitness tracking device readings via mail or Qualtrics.

### 2.3 Intervention

Extension educators have historically delivered informal education and practical skills to youth and adults within their areas. In this project, a local Extension educator in each community received training to serve as a Change Club facilitator. These facilitators led stepwise planning meetings and guided group members through lessons on civic engagement and PSE change using in-person, online, or hybrid meeting formats. Training included a comprehensive overview of the Change Club curriculum and the facilitator guide, ensuring coverage of all content modules. After their training, these facilitators conducted the first set of Change Club modules and continued to meet with and support their groups throughout the project.

The intervention is explained in detail in a separate publication (51) and summarized here. The curriculum consisted of 24 content modules that were originally conceived to be delivered in weekly meetings of approximately 1 h over 6 months. To help fulfill study aims under new constraints imposed by the pandemic, the implementation protocol was adapted to allow flexibility in the mode of delivery (in-person, virtual, or hybrid) and in meeting length and frequency. Fidelity to the curriculum was assessed using post-meeting surveys completed by facilitators (51). These data show that most meetings were delivered in person or in a hybrid format, and that three communities completed the curriculum within 6 months, while another three took over 10 months with substantial gaps between modules (121).

The initial modules focused on helping participants build group cohesion and a shared identity and establishing group guidelines. Later meetings centered on pinpointing community needs and making action plans; CCM conducted an assessment of community assets (59), explored a list of feasible changes to the built environment, and selected one or more that could be realistically achieved within 6 months. Potential interventions were chosen from options that: (1) were recommended by the Community Preventive Services Task Force (60); (2) received a Class I or II rating from the American Heart Association, indicating substantial evidence for improving diet or PA at the population level (61); and/or (3) appeared in the Global Action Plan for Noncommunicable Diseases Prevention and Control (62). After those meetings, CCM were provided with access to online modules on nutrition and PA that focused on understanding social and environmental influences. These online modules were shown during the group meetings, and then facilitators encouraged CCM to discuss new insights and explain how they were applying healthy habits in their daily lives.

At the conclusion of data collection (36 months after baseline), the control communities will be provided with intervention materials, but their outcomes will not be measured (51).

### 2.4 Outcomes

All outcomes were self-reported at baseline and approximately 2 years later. All scales were calculated as means of items and ranged from 1 to 5 unless otherwise noted.

The primary outcome was the LS7 composite cardiovascular health score, which includes BMI, blood pressure, total cholesterol, blood sugar, smoking, diet, and PA (63) categorized into poor (0), intermediate (1), or ideal (2) (see Table 1), and then summed for a total LS7 score between 0 and 14 (with higher scores indicating better health). Because there was a high volume of missing data for cholesterol and blood sugar, we also calculated a 5-component score that excluded these two items for use in sensitivity analyses.

**Table 1:** Life’s simple 7 components, survey questions, and scoring.

Component		Poor		Intermediate		Ideal	
**Body mass index**		≥30		25– < 30		<25	
**Blood pressure**				Not poor	and	Not poor or intermediate	and
“…diagnosed by a healthcare provider as having. coronary heart disease/chest pain, heart attack, heart failure, stroke/transient ischemic attack (TIA), vascular disease, or congenital heart defects”		Yes	or				
“…told by a healthcare provider that your most recent blood pressure level was:”		High	or	Elevated	or	Normal	or
“…take medication to lower their blood pressure”				Yes	or		
Reported systolic value (mmHg)		≥140	or	120–139	or	<120	and
Reported diastolic value (mmHg)		≥90		80–89		<80	
**Cholesterol**				Not poor	and	Not poor or intermediate	and
“…told by a healthcare provider that. total cholesterol level was”:		High	or	Borderline high	or	Normal	or
“…take medication to lower cholesterol”				Yes	or		
Reported total cholesterol (mg/dL)		≥240		200–239		<200	
**Blood glucose**				Not poor	and	Not poor or intermediate	and
“…told by a healthcare provider that fasting blood glucose level indicated:”		Diabetes	or	Pre-diabetes	or	Normal	or
“…take medication to lower blood sugar”				Yes	or		
Reported fasting blood glucose (mg/dL)		≥126		100–125		<100	
**Smoking**		Currently		Quit ≤1 year ago		Never or quit >1 year ago	
**Diet (# of ideal behaviors)**							
Beverages with added sugar (<36 oz./week)		0 or 1		2 or 3		4 or 5	
Fish (≥2 servings/week)							
Fruit	(≥4.5 cups fruit + vegetables/day)						
Vegetables							
Whole grains (≥3.5 servings/day)							
Sodium habits (≥2 habits)							
“…avoided prepackaged & processed foods”							
“…rarely ate out or sought lower sodium options”							
“…avoided adding salt when cooking”							
**Physical activity (minutes/week)**							
Total moderate		0	and	1–149	or	≥150	or
Total vigorous		0		1–74		≥75	

CCM were asked to follow standard anthropometric measurement procedures that instruct participants to weigh themselves twice without clothes or shoes and to report each body weight measurement in pounds (and a third time if measures differed by more than ½ pound) (64). CCM also measured their height in feet and inches or centimeters. CCM were provided with a body weight scale and a soft tape measure (if unavailable). BMI was calculated from the mean of the closest two weight measurements and height (65). Five questions were asked about blood pressure status, three about cholesterol, three about blood glucose, and one about smoking to categorize these components. CCM were asked eight LS7 questions about six types of food to categorize the diet components (63). CCM also completed the International Physical Activity Questionnaire (IPAQ-long), which includes 25 questions on PA time related to employment, transportation, domestic tasks, and leisure, as well as exercise intensities (moderate, vigorous, walking) (66). Two questions about sitting were not included. Responses were used to estimate minutes per week of moderate and vigorous PA using the recommended scoring procedures (67, 68).

Secondary outcomes included the 2018 World Cancer Research Fund/American Institute for Cancer Research (WCRF/AICR) composite recommendation adherence scale (69). The WCRF/AICR scale was based on reports related to seven recommendations: be a healthy weight; be physically active; eat a diet rich in whole grains, vegetables, fruits, and beans; and limit consumption of ultra-processed foods (UPFs), red and processed meats, sugar-sweetened beverages, and alcohol. Each component was scored according to National Cancer Institute (NCI) scoring guidelines (70) as not meeting recommendation (0), partially meeting (0.5), or meeting (1), except for the recommendation to eat a diet rich in whole grains, vegetables, fruits, and beans which was assessed by two variables (FV and fiber) each of which contributed half the points (see Table 2).

**Table 2:** World Cancer Research Fund/American Institute for Cancer Research (WCRF/AICR) scale components, survey questions, and categorization.

Recommendation	Variable	Not meeting		Partially meeting		Meeting	
Be a healthy weight	Body mass index	<18.5 or ≥30		25 – < 30		18.5 – < 25	
Be physically active	Moderate + vigorous physical activity (minutes/week)	<75		75 – < 150		≥150	
Eat a diet rich in whole grains, vegetables, fruits, and beans	Total fruit and vegetable intake (cup equivalents/day)	<1.25		1.25 – < 2.5		≥2.5	
	Total fiber intake (g/day)	<15		15 – < 30		≥30	
Limit ultra-processed foods	Frequency tertile (times/day)	≥1.56		0.75 – < 1.56		<0.75	
Limit red and processed meats	Red meat (g/week)	>500	or	≤500	and	≤500	and
	Processed meat (g/week)	≥100		21 – < 100		<21	
Limit sugar-sweetened beverages	Sugar-sweetened beverages (g/day)	>250		>0 – ≤ 250		0	
Limit alcohol	Alcohol (drinks/day)	Men: >2 Women: >1		Men: 1–2 Women: 1		0	

The six variables used to assess the WCRF/AICR diet components were created from responses to 26 frequency questions from the NCI Dietary Screening Questionnaire (DSQ) (71). Total FV intake (cup equivalents/day) was estimated using frequency of consumption of nine DSQ items [fruit, 100% fruit juice, salad, potatoes (excluding fried), dried beans, other vegetables, pizza, salsa, and tomato sauce] and the sex- and age-specific scoring algorithms provided by the NCI (72). Total fiber intake (g/day) was calculated using the frequencies of all items except for red and processed meat and the currently recommended NCI sex- and age-specific scoring algorithms (72, 73). We adapted the open-ended DSQ questions regarding cereal type (used to create fiber content tertiles) into one question about “…kind of cereal did you usually eat?” with response options of “cereals like…” (1) “puffed rice, unsweetened oatmeal, or cream of wheat,” (2) “corn flakes, Rice Chex™, or Muesli,” or (3) “corn pops, fruit loops, or Cocoa Puffs™.” Cereal frequency was included in the total fiber calculation for those who selected category 1 cereals (the highest-fiber category). UPF consumption (times/day) was calculated as the sum of frequencies of consuming fried potatoes, cookies/cake/pie/brownies, doughnuts, frozen desserts, candy, pizza, and processed meat. For CCM, who usually consume cereal in the third category (lowest fiber), cereal was classified as an UPF. The total frequency of consuming UPFs was divided into tertiles, as recommended (74), based on National Health and Nutrition Examination Survey (NHANES) 2009-10 (75) median daily frequency of consumption. CCM were also asked about the frequency of red and processed meat consumption. Average portion sizes of each (g/occasion) were estimated from median daily grams in a U.S. sample (76) divided by median daily consumption frequency from NHANES 2009-10 (75). Reported daily frequencies were multiplied by 7, and the resulting estimated portion size was used to calculate the total consumption of each (g/week). Total sugar-sweetened beverage intake was assessed with three DSQ items (regular soda or pop, coffee or tea with added sugar, and sweetened fruit, sports, or energy drinks). If any were reported, NCI sex- and age-specific scoring algorithms were used to estimate daily teaspoon equivalents of added sugar (72), which was converted to grams of sugar-sweetened beverages (77, 78). Two questions were adapted from the Alcohol-Use Disorders Identification Test (AUDIT) tool to assess (1) frequency of having a drink containing alcohol and (2) typical number of drinks containing alcohol on a typical day when drinking. Responses to these questions were used to calculate the mean number of drinks per day.

Secondary individual outcomes included separate reporting of all LS7 and WCRF/AICR components described above: BMI, high/elevated blood pressure, high/borderline high cholesterol, diabetes/pre-diabetes, current smoker, total FV intake (cups/day), total whole grain consumption (servings/day), total fiber (g/day), met the recommendation for fish intake, frequency of consuming UPFs (times/month), red and processed meat consumption (g/week), alcohol consumption (drinks/day), and total PA (MET-min/week). Additional individual outcomes included general health status measured by the SF-36 general health question (79) and transformed into an indicator of ‘fair/poor health,’ and waist circumference, which was measured twice in inches (and a third time if the measurements differed by more than ¼ inch), and the two closest measurements were averaged.

Participants were also asked to provide alternate measures of eating and PA behaviors. They completed a single 24-h recall via the Automated Self-Administered 24-h Dietary Assessment Tool (ASA24) (80). Version 2020 was used at baseline, and version 2024 at the 24-month follow-up. All recalls were examined for completeness and accuracy in accordance with the NCI scoring documentation (81). Seven measures were extracted from ASA24 data: total FV intake (cups/day), consumption of whole grains (servings/day), fiber intake (g/day), consumption of UPFs (% total kcal), red and processed meat consumption (g/week), alcohol consumption (drinks/day), and total Healthy Eating Index (HEI) score. Total FV intake (cups/day) was calculated by summing total vegetables (total dark green, red and orange, starchy, and other vegetables, but excluding legumes) and total fruit (total whole or cut intact fruits and fruit juices). Total daily ounce equivalents of meat (beef, veal, pork, lamb, and game meat, excluding organ meat and cured meat) and cured meat (frankfurters, sausages, corned beef, and luncheon meat that are made from beef, pork, or poultry) were added together, converted to grams, and multiplied by seven to estimate g/week of red and processed meat consumption. UPFs were estimated using a standard approach developed by NCI to link Nova Food Classification System (Nova) groups with foods reported in the 24-h recall by Food and Nutrient Database for Dietary Studies food codes (82, 83). We considered food items to be UPFs if they were classified by NCI as entirely Nova group 4; Nova group 4 refers to the most processed foods. The kcals from all UPFs were summed and divided by the total kcal to estimate the total daily consumption of UPF (% kcal). The total HEI score was calculated using the HEI scoring algorithm (84) and SAS code developed by NCI (85). Participants were also asked to wear a provided pedometer or an approved fitness-tracking device for 7 days, record on a paper log, and report their step count for each day. Total steps per day was the mean of up to 7 days of self-reported step counts for participants who reported at least 3 days of steps.

Other individual outcomes included mean scale measures of motivation, confidence, and social support for healthy eating and exercise, adapted from existing tools (see Table 3).

**Table 3:** Adapted scales of attitudes, self-efficacy, and social support.

Adapted scales	Items	Range of responses
Healthy eating motivation (113)	“It is important that the food I eat is nutritious” “I always follow a healthy and balanced diet” “The healthiness of food has little impact on my food choices” *(reversed)*	1-Strongly disagree 5-Strongly agree
Healthy eating habits confidence (114–116)	Confidence in… ‘eating fruits and/or vegetables at most meals’ ‘eating a variety of vegetables from all subgroups’ ‘eating more 100% whole grain foods’ ‘eating more plant-based proteins’ ‘minimizing intake of foods and beverages that are high in added sugars’	1-Not at all confident 5-Completely confident
Social support from family for healthy eating (29)	How often family/household members… ‘had eaten healthy foods (like fruits, vegetables, whole grains) with you’ ‘encouraged you to eat healthy foods’ ‘discouraged you from eating less healthy foods’	1-Never 5-Very often Not Applicable (*recode to never*)
Social support from friends for healthy eating (29)	How often friends/colleagues… ‘had eaten healthy foods (like fruits, vegetables, whole grains) with you’ ‘encouraged you to eat healthy foods’ ‘discouraged you from eating less healthy foods’	1-Never 5-Very often Not Applicable (*recode to never*)
Exercise attitudes (117)	‘It is hard for me to fit exercise into my life’ *(reversed)* ‘I exercise because it is good for my health’ ‘Exercise makes me feel better’ ‘I cannot exercise’ *(reversed)*	1-Strongly disagree 5-Strongly agree
Exercise Confidence (116)	How confident to… ‘set aside time for a physical activity program… at least 30 min, 3 times a week’ ‘stick to your exercise program when you are busy’ ‘stick to your exercise program when you feel stressed’	1-Not at all confident 5-Completely confident
Social support from family for physical activity (29)	How often family/household members… ‘participated in physical activity or exercise with you’ ‘encouraged you to be physically active’ ‘discouraged you from sitting around too much’	1-Never 5-Very often Not Applicable (*recode to never*)
Social support from friends for physical activity (29)	How often friends/colleagues… ‘participated in physical activity or exercise with you’ ‘encouraged you to be physically active’ ‘discouraged you from sitting around too much’	1-Never 5-Very often Not Applicable (*recode to never*)

Cluster-level outcomes included social engagement and cohesion, civic engagement attitudes and behaviors, an orientation toward a culture of health, and measures of the community food and PA environments. Social engagement was directly assessed by the validated Lubben Social Network Scale (86), Individual Mobilization by Jake and Shannon’s Individual Mobilization-Human Capital subscale (87), and Investment in Community Health by the Robert Wood Johnson Foundation National Survey of Health Attitudes (88). All other measures were adapted from other tools (see Table 4).

**Table 4:** Adapted measures of cluster-level outcomes.

Adapted scales	Items	Range of responses
Lubben social network (86)	*No adaptations*	
(Community) social cohesion (118)	People in my community… ‘are willing to help their neighbors’ ‘generally get along with each other’ ‘can be trusted’ ‘share the same values’	1-Strongly disagree 5-Strongly agree
Individual mobilization-human capital (87)	*No adaptations*	
Civic engagement attitudes (119)	‘I feel responsible for my community’ ‘I believe I should make a difference in my community’ ‘I believe that I have a responsibility to help those in need’ ‘I am committed to serving in my community’ ‘I believe that all people have a responsibility to their community’ ‘I believe that it is important to be informed of community issues’ ‘I believe that it is important to volunteer’ ‘I believe that it is important to financially support charitable organizations.’	1-Strongly disagree 5-Strongly agree
Civic engagement behaviors (119)	‘I am involved in regular volunteer position(s) in my community’ ‘When working with others, I make positive changes in my community’ ‘I help members of my community’ ‘I stay informed of events in my community’ ‘I participate in discussions that raise issues of social responsibility’ ‘I contribute to charitable organizations within my community’	1-Never 5-Always
Investment in community health (88)	*No adaptations*	
Fresh fruit and vegetable availability (120)	‘It is easy to buy fresh fruits and vegetables in my community’ ‘The fresh produce in my community is of high quality’ ‘There is a large selection of fresh fruits and vegetables in my community’	1-Strongly disagree 5-Strongly agree
Store selection motivation (120)	How important are… in your decision to shop at the store where you buy most of your food: Selection of foods Quality of foods Prices of foods	1-Not at all important 5-Very important
Restaurant healthy food availability (120)	‘There are many healthy menu options available’ ‘It is difficult to find healthy options when eating out, including fruit and vegetable choices’ *(reversed)* ‘It is easy to find healthy fruit and vegetable choices at the restaurants where you go most often’	1-Strongly disagree 5-Strongly agree
Walking environment (118)	‘My community offers many opportunities to be physically active’ ‘Facilities in my community offer many opportunities to get exercise’ ‘It is pleasant to walk in my community’ ‘The trees in my community provide enough shade’ ‘In my community it is easy to walk places’ ‘I often see other people walking in my community’ ‘I often see other people exercising in my community’	1-Strongly disagree 5-Strongly agree
Community safety (118)	‘I feel safe walking in my community, day or night’ ‘Violence is not a problem in my community’ ‘My community is safe from crime.’	1-Strongly disagree 5-Strongly agree
Community aesthetic quality (118)	‘There is a lot of trash and litter on the streets in my community’ ‘There is a lot of noise in my community’	1-Strongly disagree 5-Strongly agree

### 2.5 Sample size

Sample size targets for CCM were based on the power to detect a significant difference between the intervention and control groups at two-year follow-up using two-sided *t*-tests with 80% power and an alpha level of 0.05. In other interventions that included a Change Club component, we observed effect sizes of +0.7–0.8 units on the LS7 composite score (89, 90). We assumed consistent cluster sizes across communities, 10% annual attrition, and an intraclass correlation within clusters of 0.025. Under these conditions, we aimed to recruit 140 participants (70 per arm), yielding an effective sample size of 45 CCM per arm at 2-year follow-up to detect an effect size of 0.6 SDs (approximately 1 unit in the LS7 composite score). Our sample of 196 CCM exceeds this target.

### 2.6 Randomization

Communities were paired within state (New York and Texas) using population and rurality based on RUCA version 2.0 (54). Research staff grouped communities into matched pairs and generated one random number for each pair. If the number was odd, the first community in the pair was assigned to the intervention; if even, the second community was assigned to the intervention. Research staff communicated assignments to Extension staff who delivered the curriculum. Assignment of communities could not be concealed from CCM or research staff due to the nature of the study design; however, field staff involved in intervention delivery were not involved in assessing any outcomes (51).

### 2.7 Missing data

We report on basic reporting standards for missing data (91). At baseline, 137 CCM provided complete case survey data. At year-2 follow-up, 99 CCM provided complete case survey data (50.5%), 46 cases were missing (attrition of 23.5%), and 51 were missing one or more variables (26.0%). Whether a participant had diabetes or pre-diabetes was the outcome most often missing at baseline (17.3%) and at year-2 (37.2%). Complete case data across baseline and year-2 were available for 79 (40.3%) of 196 CCM. Survey outcomes for cases missing data at year-2 and those not missing were compared using *t*-tests and Chi-square analysis to examine the pattern of missingness. Significant differences were observed in 13 of 37 outcomes (35.1%), but no consistent directionality was observed. CCM missing year-2 data were “healthier” on eight outcomes and “less healthy” on five outcomes, which does not provide strong evidence of systematic bias due to missing data.

Multiple imputation using fully conditional specification (FCS) (92) was applied to address missing data in all samples (93–96). Multiple imputation is the most accurate approach to estimating missing data and standard errors that account for both variability due to sampling and to imputation itself (93), and it performs well even in small samples and with up to half of the data missing (97). FCS is considered an unbiased and robust approach to the imputation of missing continuous and classification data (95). Thirty imputations (93, 98, 99) were generated using a model that included all survey outcome variables (baseline and year-2 follow-up), baseline characteristics with missing observations (relationship status and household income), all design variables (pair, study arm), and all year-1 outcomes and remaining baseline characteristics (age, sex [men or not], race, ethnicity, employment, education, number of household adults, and number of household children) as auxiliary variables (93, 94, 100). We used the default number of burn-in iterations, which is 20 (101). To avoid bias, composite variables were included in the model rather than recalculating them after imputation (98). Outcome measures derived from 24-h recall and pedometry were imputed separately following the same procedures, with survey measures of diet and PA serving as auxiliary variables.

Discriminant function and linear regression were used for categorical and continuous outcomes, respectively [PROC MI, SAS software (SAS Institute Inc., Cary, NC, USA; version 9.4)]. We confirmed that convergence was achieved by examining trace plots and observed no trends over the 30 imputations.

### 2.8 Analysis

Baseline sample characteristics by study arm were tabulated for CCM to describe comparability of intervention and control groups.

Normality of continuous outcomes was checked by examining histograms and checking skewness statistics. Variables with skewness greater than +/−2 (102) were examined in boxplots, and outliers were identified as greater than 3 IQR above Q3 or below Q1 with a discernible break in the distribution. Outliers were flagged for sensitivity analyses when the primary analyses showed significant effects, but none of the results met these criteria.

Net effects of the intervention across study arms were estimated for each outcome using an ITT framework that compared all participants assigned to the intervention to all controls (52). Secondarily, per-protocol net effects were estimated by comparing the sub-sample of CCM attendees (who attended at least one Change Club activity) to all controls (53). Multivariate linear regression was used to assess the net effects of the intervention on year-2 values of the primary outcome and continuous secondary outcomes (PROC MIXED, SAS software). Multivariate logistic regression was used to assess the net effects of the intervention on year-2 values of binary secondary outcomes (PROC GLIMMIX, SAS software). Both types of models included the study arm and matched pair as fixed effects, community as a random effect (to account for community clustering), and controlled for the baseline value of the outcome being assessed. Fixed effects were tested using F-tests with the Kenward-Roger degrees-of-freedom approximation. The same models estimated adjusted means for continuous outcomes and predicted probabilities for binary outcomes at year-2. Regressions were performed separately on each imputed dataset, and results were combined (PROC MIANALYZE, SAS software).

Identical linear regression models for the LS7 calculation, excluding cholesterol and blood sugar (as described above), were performed as sensitivity analyses.

Associations between intervention dose and year-2 outcomes were also explored within the intervention arm (*n* = 90). First, associations between any attendance and year-2 outcomes were examined using multivariate linear and logistic regression models in which an indicator of any attendance replaced the study-arm indicator. Second, dose–response associations were examined in a subsample of attendees (*n* = 55) using regression models in which the number of meetings attended replaced the study-arm indicator. In both analyses, the models controlled for the baseline value of the outcome being assessed and included community as a random effect.

## 3 Results

**Figure 1:**
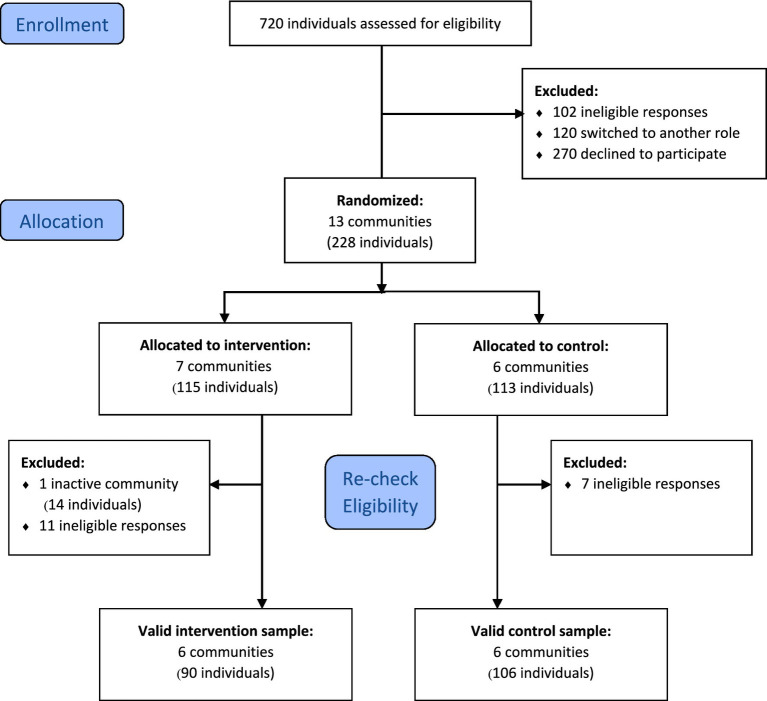
Change Club Member enrollment flowchart.

In total, 720 individuals in 13 communities were screened for CCM eligibility, and 618 were deemed eligible (85.8%; Figure 1). Among eligible individuals, 120 individuals chose not to participate as CCM but switched to the community resident role (19.4%), 270 declined to participate at all (43.7%), and 228 individuals consented to participate as CCM (36.9%). Seven communities were allocated to the intervention arm and six communities to the control. Approximately 1 year after consenting, community and individual eligibility were confirmed, and one community and 32 individuals were excluded from the sample. The final analytic sample included six communities in the intervention arm (*n* = 90) and six communities in the control arm (*n* = 106).

CCMs were typically women (74.5%), aged 45 to 64 years (41.3%), white (82.1%), and non-Hispanic (88.8%; Table 5). Most had earned a college degree (33.7%) or more education (20.9%), were employed (57.7%), and were married (62.6%). Most households had two adults (62.8%), and most had no children living at home (66.8%). Household incomes varied widely, and most were food secure (60.2%) and received no government assistance (80.5%). About half of households were in TX (43.4%) and half in NY (56.6%). Participant characteristics appeared consistent across study arms.

**Table 5:** Baseline participant and household characteristics by study arm.

Characteristics		Total		Participants in intervention communities			Participants in control communities		
		*n*	%	*n*	Count	%	*n*	Count	%
Individual characteristics									
Sex	Men	196	25.5	90	27	30.0	106	23	21.7
	Women		74.5		63	70.0		83	78.3
	Not one of the above		0.0		0	0.0		0	0.0
	Prefer not to answer		0.0		0	0.0		0	0.0
Age	18 to 24 years	196	2.0	90	1	1.1	106	3	2.8
	25 to 34 years		14.3		10	11.1		18	17.0
	35 to 44 years		19.9		21	23.3		18	17.0
	45 to 64 years		41.3		38	42.2		43	40.6
	65 years and older		22.4		20	22.2		24	22.6
Race	American Indian/Alaska Native	196	3.6	90	2	2.2	106	5	4.7
	Asian		0.5		1	1.1		0	0.0
	Native Hawaiian/Pacific Islander		0.5		1	1.1		0	0.0
	Black		9.7		7	7.8		12	11.3
	White		82.1		76	84.4		85	80.2
	More than one race		0.5		0	0.0		1	0.9
	Not one of the above		3.1		3	3.3		3	2.8
	Prefer not to answer		0.0		0	0.0		0	0.0
Hispanic	Yes	196	11.2	90	10	11.1	106	12	11.3
Highest year of school	High school or less	196	18.9	90	19	21.1	106	18	17.0
	Technical or vocational school		2.0		2	2.2		2	1.9
	Some college		24.5		14	15.6		34	32.1
	College graduate		33.7		31	34.4		35	33.0
	Graduate or professional degree		20.9		24	26.7		17	16.0
Employment status	Employed	196	57.7	90	55	61.1	106	58	54.7
	Out of work		6.1		8	8.9		4	3.8
	A homemaker		3.6		3	3.3		4	3.8
	Student/retired		26.0		18	20.0		33	31.1
	Unable to work		6.6		6	6.7		7	6.6
Marital status	Married	195	62.6	90	59	65.6	105	63	60.0
	Separated, divorced, or widowed		15.9		16	17.8		15	14.3
	Never married		12.3		8	8.9		16	15.2
	A member of an unmarried couple		9.2		7	7.8		11	10.5
Household characteristics									
# Adults in household	1 Adult	196	18.9	90	17	18.9	106	20	18.9
	2 Adults		62.8		58	64.4		65	61.3
	3 Or more adults		18.4		15	16.7		21	19.8
# Children in the household	0 Children	196	66.8	90	55	61.1	106	76	71.7
	1 Child		14.3		19	21.1		9	8.5
	2 Children		9.2		8	8.9		10	9.4
	3 Children		6.1		8	8.9		4	3.8
	4 Or more children		3.6		0	0.0		7	6.6
Annual household income	Less than $10,000	193	6.2	90	8	8.9	103	4	3.9
	$10,000–$14,999		4.7		6	6.7		3	2.9
	$15,000–$19,999		7.3		6	6.7		8	7.8
	$20,000–$24,999		3.1		2	2.2		4	3.9
	$25,000–$34,999		9.3		7	7.8		11	10.7
	$35,000–$49,999		19.2		15	16.7		22	21.4
	$50,000–$74,999		16.6		9	10.0		23	22.3
	$75,000 or more		33.7		37	41.1		28	27.2
Household is food secure (%)	Food secure	196	60.2	90	58	64.4	106	60	56.6
	Food insecure		39.8		32	35.6		46	43.4
Any government assistance received (%)		195	19.5	90	17	18.9	105	21	20.0
State	Texas	196	43.4	90	40	44.4	106	45	42.5
	New York		56.6		50	55.6		61	57.5

Using the ITT framework, we observed no significant difference in the LS7 composite at year-2 among individuals in the intervention arm relative to control, adjusted for baseline values (+0.01, *p* = 0.971; Table 6), nor in sensitivity analysis of the alternate LS7 calculation that excluded cholesterol and blood sugar (+0.03, *p* = 0.930). There were also no significant between-group differences in the year-2 secondary individual outcomes after adjustment for baseline values. Among the 12 collective and environmental outcomes examined, there was one significant net effect of intervention on year-2 store selection motivation (−0.20, *p* = 0.046; Table 7). Using a per-protocol approach, no significant differences were observed (Supplementary Tables 1, 2, column 1).

**Table 6:** Two-year Intervention effects on individual outcomes among Change Club Members.

Outcomes	Intervention (*n* = 90)	Control (*n* = 106)	Adjusted net impact	Adjusted sig.
	Year-2 adj. mean/%	Year-2 adj. mean/%		
Primary outcome				
Simple 7 cardiovascular health score (0–14)	8.92	8.91	0.01	0.971
Secondary outcomes				
Health outcomes				
Body mass index	30.68	31.17	−0.48	0.478
Waist circumference (inches)	38.98	39.24	−0.26	0.805
High/elevated blood pressure (%)	62.5	63.2	OR = 0.97	0.963
High/borderline total cholesterol (%)	57.0	54.1	OR = 1.12	0.793
Diabetes/pre-diabetes (%)	35.4	33.4	OR = 1.09	0.874
Fair/poor health (%)	31.2	39.0	OR = 0.71	0.614
Current smoker (%)^^^	22.4	31.9	OR = 0.62	0.632
World Cancer Research Fund/American Institute for Cancer Research cancer recommendation composite score (0–7)	3.40	3.37	+0.03	0.842
Eating behaviors (survey)				
Total fruit and vegetable consumption (cups/day)	2.58	2.66	−0.08	0.725
Total whole grain consumption (servings/day)	1.05	0.99	+0.06	0.699
Total fiber (g/day)	15.63	15.17	+0.47	0.240
Met recommendation for fish (%)	22.2	26.8	OR = 0.78	0.591
Frequency of consuming ultra-processed foods (times/month)	39.56	39.95	−0.38	0.939
Red and processed meat consumption (g/week)	1019.43	1131.96	−112.53	0.427
Alcohol consumption (drinks/day)	0.21	0.23	−0.02	0.796
Eating behaviors (24-h recall)				
Total Healthy Eating Index score	49.40	50.53	−1.13	0.747
Total fruit and vegetable consumption (cups/day)	2.40	2.70	−0.31	0.605
Total whole grain consumption (servings/day)	0.83	0.88	−0.04	0.924
Total fiber (g/day)	15.66	16.22	−0.56	0.837
Ultra-processed foods consumption (%kcal)	51.54	49.28	+2.26	0.764
Red and processed meat consumption (g/week)	548.48	619.61	−71.13	0.799
Alcohol consumption (drinks/day)	0.47	0.77	−0.30	0.612
Physical activity behaviors				
Total physical activity (MET-min/week)	1439.93	1445.90	−5.97	0.962
Total steps per day (pedometry)	5745.23	6180.70	−435.47	0.503
Attitudes, self-efficacy, and social support				
**For healthy eating**				
Healthy eating motivation scale (1–5)	3.67	3.77	−0.10	0.254
Healthy eating habits confidence scale (1–5)	3.31	3.35	−0.04	0.779
Social support from family for healthy eating scale (1–5)	3.20	3.17	+0.03	0.858
Social support from friends for healthy eating scale (1–5)	2.60	2.67	−0.07	0.653
**For physical activity**				
Exercise attitudes scale (1–5)	3.63	3.77	−0.14	0.194
Exercise confidence scale (1–5)	3.09	3.17	−0.08	0.677
Social support from family for physical activity scale (1–5)	2.38	2.51	−0.13	0.396
Social support from friends for physical activity scale (1–5)	2.07	2.21	−0.14	0.393

MET, metabolic equivalent of task.

All analyses of change in individual outcomes used an intent-to-treat framework and multiply imputed data.

Adjusted means/predicted probabilities at year-2 were estimated, and the adjusted net intervention effects on the outcome values at year-2 were tested, using multiple linear regression for continuous variables and multiple logistic regression for dichotomous variables controlling for random assignment pair, baseline value of the outcome, and community.

^One pair was excluded because it had too few smokers for the model to converge.

**Table 7:** Two-year intervention effects on collective and environmental outcomes among Change Club Members.

Outcomes	Intervention (*n* = 90)	Control (*n* = 106)	Adjusted net impact	Adjusted sig.
Year-2 adj. mean	Year-2 adj. mean			
Social outcomes				
Social capital and cohesion				
Social engagement *(family & friends)* scale (0–5)	2.62	2.62	−0.00	0.993
*(Community)* social cohesion scale (1–5)	3.57	3.46	+0.12	0.290
Civic engagement				
Individual mobilization-human capital subscale (1–5)	3.39	3.47	−0.07	0.328
General civic engagement attitudes scale (1–5)	3.96	3.95	+0.01	0.937
General civic engagement behaviors scale (1–5)	3.23	3.27	−0.04	0.714
Culture of health				
Investment in community health (# of high priorities; 0–5)	3.82	4.07	−0.25	0.228
Environment outcomes				
Community food environment				
Fresh fruit and vegetable availability scale (1–5)	3.41	3.53	−0.11	0.573
Store selection motivation scale (1–5)	4.38	4.58	−0.20	**0.046**
Restaurant healthy food availability scale (1–5)	3.23	3.25	−0.02	0.866
Community physical activity environment				
Walking environment scale (1–5)	3.22	3.27	−0.04	0.743
Community safety scale (1–5)	3.26	3.18	+0.09	0.488
Community aesthetic quality scale (1–5)	3.22	3.28	−0.06	0.643

All analyses of change in collective and environmental outcomes used an intent-to-treat framework and multiply imputed data.

Adjusted means at year-2 were estimated, and the net intervention effects on the outcome values at year-2 were tested, using multiple linear regression controlling for random assignment pair, baseline value of the outcome, and community.

Bold *p*-value indicates significance at a 95% confidence level.

Thirty-five CCMs never attended any Change Club meetings (38.9%). In exploratory analyses within the intervention arm, any attendance was associated with higher LS7 composite scores (+0.93, *p* = 0.006), WCRF/AICR composite scores (+0.54, *p* = 0.008), and healthy eating motivation (+0.27, *p* = 0.028) relative to non-attendees, after adjusting for baseline outcome value (Supplementary Table 1, column 2). No differences were observed for collective or environment outcomes among attendees relative to non-attendees (Supplementary Table 2, column 2). Among attendees, no dose–response pattern was observed between the count of meetings attended and any year-2 outcome, with adjustment for baseline outcome value (Supplementary Table 1, 2, column 3).

## 4 Discussion

In this cRCT in which CCM worked together to make PSE changes in their rural communities to make it easier to be more physically active and/or eat more healthfully, there were no significant differences between participants in the intervention and control arms on the LS7 composite score or any secondary individual outcomes at year-2. Among the collective outcomes, there was only one significant difference between arms (which may be explained by chance). Interestingly, however, nearly 40% of CCM never attended any Change Club meetings, and those who did had significantly higher year-2 LS7 composite scores, WCRF/AICR composite scores, and healthy eating motivation, compared to non-attendees (slightly more than expected by chance alone).

These findings provide important context with consideration of prior research. While there is a theoretical basis for civic engagement interventions to improve individual-level health outcomes, there is no well-established empirical evidence for this. Within the one pilot study of a civic engagement intervention that measured individual-level health outcome changes, this cultural adaptation of the Change Club curriculum with African American women in Boston churches resulted in significant improvements in CCMs’ waist circumference, cardiovascular fitness, and blood pressure (38). However, the Boston pilot differed from this study in three important ways. First, that curriculum adaptation combined the Change Club curriculum with a CVD prevention program that covered nutrition and PA topics during each meeting (38). In the current study, CCM viewed online modules in group meetings on nutrition and PA, but these were not covered in the same format or level of detail as the Boston study. This study’s ongoing process evaluation (which will be published elsewhere) suggests that the civic engagement aspects of the curriculum were implemented with high fidelity, whereas online diet and PA module usage showed much greater variability and were implemented with much lower fidelity. Second, the Boston pilot intervention lasted just 6 months, with post-intervention data collection occurring closely after the last session. The short duration of that intervention and the proximity of data collection may have contributed to the observed improvements in individual-level health outcomes. Conversely, the Change Clubs in this study met for a longer period (10 months on average) with some gaps between meetings, and data collection occurred annually rather than aligned with a particular point in intervention implementation. Third, eligibility criteria may have resulted in the enrollment of less healthy individuals in the Boston study, which left room for greater health improvements. In the Boston study, inclusion criteria included BMI ≥ 25 and being currently sedentary (38), whereas the present study only required CCM to score “poor” or “intermediate” on at least one of the LS7 composite score items. This could mean, for example, that a participant could score ‘intermediate’ in the smoking component (‘former smoker, quit >1 year ago’) only (or any other single category) and score ‘ideal’ for all other components (BMI, diet, PA, blood pressure, cholesterol, and blood glucose) and still be eligible to enroll as a CCM in the current study.

Other studies that suggested civic engagement interventions were positively associated with PA among residents or park users did not measure PA among the people who were themselves civically engaged. For example, one study found that involvement of community groups in playground design, selection, installation, and maintenance was associated with differential increases in the number of people participating in moderate to vigorous PA in the parks 12 months after playground renovation compared to parks without community group involvement (50); however, they did not collect information about PA among the involved community group members. Volunteer programs also may be classified as civic engagement (but not necessarily for PSE change). For example, the Experience Corps program in Baltimore, MD, places older volunteers in public elementary schools for 15 h per week in roles designed to improve the academic outcomes of children. A small RCT reported that after volunteering for 4–8 months, Experience Corps volunteers with low baseline activity reported increased PA relative to similar controls; whereas volunteers who were already active at baseline showed no relative change (103).

It is difficult to state definitively whether there have been more community-engaged interventions that found no significant effects (‘null reports’) during or after the COVID-19 pandemic because, in general, there is a bias against publishing null results (104). However, the pandemic posed significant challenges to community-engaged research that may have increased the likelihood of studies with non-significant findings (105). For example, many studies reported the following challenges to community-engaged research during and after the pandemic: methodological disruptions from shifting from in-person to virtual interventions and data collection visits; logistical issues by researchers and community partners that caused project delays, changes in scope, or unfinished projects; and focus pivoting to address immediate, basic community needs related to the pandemic rather than the original research objectives. A 2022 survey of National Institutes of Health-funded Principal Investigators (PIs) recruiting for interventions and trials related to health behaviors found that while 2% of PIs reported that recruitment was very difficult before COVID-19, 39% of PIs reported that recruitment was very difficult ‘now’ (in 2022). Authors reported that both recruitment and retention were challenging for a rural, longitudinal (6-month) study aimed at health care literacy; the team had to double the number of communities to meet recruitment goals, and only 52 of 127 enrolled participants (41%) completed the study (106). Some studies noted that shifting study intervention activities from in-person to virtual engagement due to COVID restrictions and guidelines led to challenges such as distractions, limited internet access, and difficulty forming relationships online (107). Other authors noted that shifting away from in-person research activities makes it difficult to engage with, recruit, and retain participants from under-resourced communities where digital infrastructure is lacking (108). Logistical issues to study interventions during the COVID era included researchers’ and community partners’ own well-being and personal circumstances, such as feeling ‘burnt out,’ needing to manage additional caretaking responsibilities, and concern about illness (107). Logistical issues also included institutional barriers such as inflexible financial, regulatory, and administrative policies (107). These COVID-19-related factors may have contributed to lower attendance and retention for CCM, as well as signing up to participate as a CCM and not fully understanding or following through on participation as a member, but only completing data collection activities.

Attendees had significantly higher LS7 composite score, WCRF/AICR composite score, and healthy eating motivation at year-2 compared to non-attendees, adjusting for baseline values. This suggests that participation in Change Clubs may lead to individual health improvements if people attend the meetings. Among attendees, the most frequently reported barrier to participation was scheduling, with childcare, transportation, medical issues, vacation, and work also mentioned [manuscript in progress]. We did not ask non-attendees about their barriers to participation and therefore cannot compare experiences for the two groups. Although the study recruitment materials explained the role of CCM, participants rarely interacted with study personnel during sign-up. Therefore, there may have been a discrepancy between what the participant believed they had signed up for and the expectations of the study. It should be acknowledged that differences between the characteristics and experiences of attendees and non-attendees (selection bias) may threaten the external validity of these results (109). Recommendations and possibilities to improve attendance in interventions like the Change Club include direct local recruitment and collaboration with existing groups, clear expectations of CCM starting at recruitment, and curriculum implementation over a shorter, more intensive duration.

Although this is discussed in detail in another study, it is worth noting that nearly 20,000 enrollment attempts (74%) were considered fraudulent. Fraudulent enrollment attempts were identified by automated checks for IP addresses outside study areas (22%), reCAPTCHA screening (10%), and active investigative procedures (34%) (57). Ongoing advances in generative artificial intelligence are expected to produce increasingly adaptive fraudulent responses. Researchers must therefore deploy flexible validation strategies that integrate automated detection with active review, calibrated to the study’s aims, population, and participant burden (57).

Strengths of this study include extensive prior research on the Change Club model, a cluster-randomized controlled design, a focus on rural areas, the inclusion of two states, and long-term follow-up. A relatively novel aspect and strength of this study is that very few studies of civic engagement for PSE change measure health outcomes among participants.

This study has limitations worth noting. One major limitation was the COVID-era timing of study recruitment. Baseline data collection for the Change Club study began in February 2020, and then needed to be paused due to the COVID-19 pandemic (110). Recruitment was initially paused, and then, when it was determined that in-person activities (e.g., data collection activities) would not be possible for the foreseeable future, the team analyzed data collection options, attempting to balance rigor, feasibility, stakeholder views, and participants’ health and safety (110). Ultimately, the decision was made to use only self-reported measurement data and validated questionnaires (110). Recruitment resumed between September 2021 and January 2022. Although vaccines were available at that time, disruptions to day-to-day life remained. For example, high rates of unemployment persisted, telework remained common for many workers, public spaces experienced intermittent closures depending on local transmission levels, and food and housing insecurity were ongoing issues. Therefore, some population subgroups may not have been willing or available to fully participate in civic engagement at that time. It is not known why some CCM did not participate in any Change Club meetings, but it is possible that the abovementioned (and other) COVID-era issues contributed. High attrition (23.5%), another limitation, was likely at least partially due to the COVID-19 era social context and may also have been due to the relatively long duration of the intervention. Comparisons of attendees and non-attendees within the intervention arm may introduce selection bias, as these individuals may differ in ways independent of the intervention; thus, the improvements in health outcomes would need to be replicated in future studies. An additional limitation is that we do not have other measures of individual-level participation; we have integrated these measures into future studies (111) because it is a crucial element that would help us better understand differences. Finally, generalizability may be limited: the CCM were primarily non-Hispanic white women who were college-educated, employed, and married; thus, results may not be representative of other groups, and the Change Club intervention should be tested with new and diverse populations. Future research studies could be conducted in more diverse communities, have bilingual recruitment materials and Change Club leaders, and promote the study via strategies that reach younger populations (e.g., TikTok).

In summary, across the full sample of intervention relative to control participants, significant net impacts on health measures, health-related behaviors, or collective outcomes were not observed. However, a notable finding is that attendees had better health-related outcomes than non-attendees, after controlling for baseline measures. These results suggest that participation and sustained involvement are critical to achieving individual-level benefits through civic engagement for PSE change interventions. The unique challenges presented by the COVID-19 pandemic, including barriers to recruitment and retention, particularly in these rural communities, may have contributed to the overall null findings and high attrition rates. Nevertheless, the study demonstrates the potential of programs like Change Clubs in promoting health among attendees. However, it also suggests that implementing interventions requires careful formative work and planning that aligns with participant and community needs and expectations, such that active attendance can be optimized, and highlights the importance of tailoring recruitment efforts, offering more intensive and time-limited interventions, and ensuring inclusivity and adaptability. Additional suggestions include recruiting group members with existing community connections and ensuring that CCMs understand the time and effort needed for PSE projects (112). Future research should aim to test tailored approaches in more diverse populations and settings to understand better how to enhance attendance and potentially improve the effectiveness of civic engagement initiatives for PSE change.

## Data Availability

De-identified raw data supporting the conclusions of this article can be made available upon request and approval via institutionally approved data sharing agreement conditions.
