# Supplementary material for: The Change Club intervention: 2-year impacts from a cluster-randomized trial in rural communities in New York and Texas
Source: Front Public Health. 2026 Jul 2;14:1793324. doi: 10.3389/fpubh.2026.1793324 (PMC13374524; doi:10.3389/fpubh.2026.1793324)
Supplement: Supplementary file 1 [file Table_1.docx]

Supplemental Table 1 for the article: *The Change Club Intervention: Two-Year Impacts from a Cluster Randomized Trial in Rural Communities in New York and Texas*

**SUPPLEMENTAL TABLE 1: Alternative Analytic Approaches for Evaluating Two-year Intervention Effects on Individual Outcomes among Change Club Members**

|  | **Analytic Approach** | | | | | |
| --- | --- | --- | --- | --- | --- | --- |
|  | **1. Per Protocol**  **(n = 161)** | | **2. Attendee vs. Not**  **(n = 90)** | | **3. Dose Response**  **(n = 55)** | |
|  | **Net Effect** | **Sig.** | **Effect** | **Sig.** | **Effect** | **Sig.** |
| **PRIMARY OUTCOME** |  |  |  |  |  |  |
| Simple 7 cardiovascular health score (0-14) | +0.42 | 0.230 | +0.93 | **0.006** | +0.00 | 0.939 |
| **SECONDARY OUTCOMES** |  |  |  |  |  |  |
| **HEALTH OUTCOMES** |  |  |  |  |  |  |
| Body mass index | -0.85 | 0.163 | -0.87 | 0.316 | -0.01 | 0.942 |
| Waist circumference (inches) | -0.42 | 0.718 | -0.35 | 0.774 | +0.15 | 0.359 |
| High/elevated blood pressure (%) | OR = 1.27 | 0.754 | OR = 1.82 | 0.297 | OR = 0.98 | 0.878 |
| High/borderline total cholesterol (%) | OR = 1.11 | 0.841 | OR = 1.06 | 0.914 | OR = 1.02 | 0.788 |
| Diabetes/pre-diabetes (%) | OR = 1.06 | 0.922 | OR = 1.34 | 0.738 | OR = 0.98 | 0.888 |
| Fair/poor health (%) | OR = 0.63 | 0.637 | OR = 1.10 | 0.905 | OR = 1.18 | 0.301 |
| Current smoker (%) | n/a^ | n/a^ | n/a^ | n/a^ | n/a^ | n/a^ |
| World Cancer Research Fund/American Institute for Cancer Research cancer recommendation composite score (0–7) | +0.24 | 0.164 | +0.54 | **0.008** | -0.01 | 0.731 |
| **EATING BEHAVIORS** (survey) |  |  |  |  |  |  |
| Total fruit and vegetable consumption (cups/day) | +0.08 | 0.794 | +0.62 | 0.067 | -0.02 | 0.666 |
| Total whole grain consumption (servings/day) | +0.17 | 0.409 | +0.23 | 0.273 | +0.00 | 0.899 |
| Total fiber (g/day) | +0.79 | 0.077 | +0.86 | 0.135 | -0.06 | 0.409 |
| Met recommendation for fish (%) | OR = 1.07 | 0.905 | OR = 2.52 | 0.246 | OR = 0.85 | 0.220 |
| Frequency of consuming ultra-processed foods (times/month) | -4.00 | 0.440 | -6.93 | 0.268 | -0.72 | 0.321 |
| Red and processed meat consumption (g/week) | -201.72 | 0.101 | -128.48 | 0.427 | +9.49 | 0.690 |
| Alcohol consumption (drinks/day) | -0.03 | 0.685 | -0.02 | 0.831 | -0.00 | 0.999 |
| **EATING BEHAVIORS** (24-hr recall) |  |  |  |  |  |  |
| Total Healthy Eating Index score | +1.55 | 0.667 | +6.10 | 0.244 | +0.59 | 0.446 |
| Total fruit and vegetable consumption (cups/day) | -0.06 | 0.928 | +0.64 | 0.451 | +0.03 | 0.818 |
| Total whole grain consumption (servings/day) | +0.22 | 0.646 | +0.55 | 0.359 | -0.00 | 0.994 |
| Total fiber (g/day) | +1.09 | 0.711 | +3.59 | 0.320 | +0.23 | 0.631 |
| Ultra-processed foods consumption (%kcal) | -2.33 | 0.766 | -11.15 | 0.303 | +1.11 | 0.410 |
| Red and processed meat consumption (g/week) | -140.05 | 0.633 | -171.74 | 0.688 | -3.46 | 0.942 |
| Alcohol consumption (drinks/day) | -0.49 | 0.460 | -0.40 | 0.533 | -0.02 | 0.822 |
| **PHYSICAL ACTIVITY BEHAVIORS** |  |  |  |  |  |  |
| Total physical activity (MET-min/week) | +62.67 | 0.733 | +126.55 | 0.543 | -7.78 | 0.782 |
| Total steps per day (pedometry) | -321.53 | 0.672 | +602.44 | 0.515 | +186.10 | 0.127 |

|  | **Analytic Approach** | | | | | |
| --- | --- | --- | --- | --- | --- | --- |
|  | **1. Per Protocol**  **(n = 161)** | | **2. Attendee vs. Not**  **(n = 90)** | | **3. Dose Response**  **(n = 55)** | |
|  | **Net Effect** | **Sig.** | **Effect** | **Sig.** | **Effect** | **Sig.** |
| **ATTITUDES, SELF-EFFICACY, AND SOCIAL SUPPORT** |  |  |  |  |  |  |
| **For Healthy Eating** |  |  |  |  |  |  |
| Healthy eating motivation scale (1-5) | +0.02 | 0.833 | +0.27 | **0.028** | -0.01 | 0.771 |
| Healthy eating habits confidence scale (1-5) | +0.11 | 0.458 | +0.25 | 0.204 | -0.00 | 0.861 |
| Social support from family for healthy eating scale (1-5) | +0.11 | 0.535 | +0.14 | 0.543 | +0.01 | 0.729 |
| Social support from friends for healthy eating scale (1-5) | -0.06 | 0.746 | +0.06 | 0.789 | -0.01 | 0.753 |
| **For Physical Activity** |  |  |  |  |  |  |
| Exercise attitudes scale (1-5) | -0.10 | 0.419 | +0.01 | 0.956 | +0.02 | 0.453 |
| Exercise confidence scale (1-5) | +0.02 | 0.916 | +0.13 | 0.625 | -0.04 | 0.258 |
| Social support from family for physical activity scale (1-5) | -0.07 | 0.704 | +0.09 | 0.671 | -0.04 | 0.230 |
| Social support from friends for physical activity scale (1-5) | -0.09 | 0.629 | +0.06 | 0.778 | -0.02 | 0.564 |
| MET: Metabolic equivalent of task  All effect sizes and p-values from multiple linear regression for continuous variables and multiple logistic regression for dichotomous variables with multiply imputed data and control for baseline value of the outcome.  Column 1: Net intervention effect on year-2 outcome for attendees relative to all controls, adjusted for random assignment pair and community.  Column 2: Effect of any attendance compared to no attendance on year-2 outcome value within intervention arm, adjusted for community.  Column 3: Dose-response effect of number of activities attended on year-2 outcome value among attendees, adjusted for community.  Bold p-value indicates significance at a 95% confidence level.  ^Too few smokers to analyze. | | | | | | |
