# Supplementary material for: The Change Club intervention: 2-year impacts from a cluster-randomized trial in rural communities in New York and Texas
Source: Front Public Health. 2026 Jul 2;14:1793324. doi: 10.3389/fpubh.2026.1793324 (PMC13374524; doi:10.3389/fpubh.2026.1793324)
Supplement: Supplementary file 2 [file Table_2.docx]

Supplemental Table 2 for the article: *The Change Club Intervention: Two-Year Impacts from a Cluster Randomized Trial in Rural Communities in New York and Texas*

**SUPPLEMENTAL TABLE 2: Alternative Analytic Approaches for Evaluating Two-year Intervention Effects on Collective and Environment Outcomes among Change Club Members**

|  | **Analytic Approach** | | | | | |
| --- | --- | --- | --- | --- | --- | --- |
|  | **1. Per Protocol**  **(n = 161)** | | **2. Attendee vs. Not**  **(n = 90)** | | **3. Dose Response**  **(n = 55)** | |
|  | **Net Effect** | **Sig.** | **Effect** | **Sig.** | **Effect** | **Sig.** |
| **SOCIAL OUTCOMES** |  |  |  |  |  |  |
| **Social Capital and Cohesion** |  |  |  |  |  |  |
| Social engagement *(family & friends)* scale (0-5) | +0.10 | 0.572 | +0.16 | 0.319 | +0.00 | 0.917 |
| *(Community)* social cohesion scale (1-5) | +0.13 | 0.297 | +0.01 | 0.922 | +0.02 | 0.310 |
| **Civic Engagement** |  |  |  |  |  |  |
| Individual mobilization-human capital subscale (1-5) | -0.03 | 0.723 | +0.12 | 0.270 | +0.02 | 0.122 |
| General civic engagement attitudes scale (1-5) | +0.06 | 0.576 | +0.13 | 0.303 | +0.02 | 0.344 |
| General civic engagement behaviors scale (1-5) | +0.02 | 0.895 | +0.12 | 0.440 | +0.03 | 0.126 |
| **Culture of Health** |  |  |  |  |  |  |
| Investment in community health (# of high priorities; 0-5) | -0.10 | 0.648 | +0.32 | 0.268 | -0.01 | 0.883 |
| **ENVIRONMENT OUTCOMES** |  |  |  |  |  |  |
| **Community Food Environment** |  |  |  |  |  |  |
| Fresh fruit and vegetable availability scale (1-5) | -0.19 | 0.553 | -0.08 | 0.730 | -0.01 | 0.672 |
| Store selection motivation scale (1-5) | -0.15 | 0.214 | +0.12 | 0.429 | -0.01 | 0.783 |
| Restaurant healthy food availability scale (1-5) | -0.06 | 0.735 | -0.11 | 0.549 | +0.02 | 0.512 |
| **Community Physical Activity Environment** |  |  |  |  |  |  |
| Walking environment scale (1-5) | -0.04 | 0.793 | -0.00 | 0.995 | -0.01 | 0.620 |
| Community safety scale (1-5) | +0.06 | 0.681 | -0.06 | 0.714 | -0.01 | 0.792 |
| Community aesthetic quality scale (1-5) | -0.04 | 0.784 | +0.06 | 0.729 | -0.00 | 0.929 |
| All effect sizes and p-values from multiple linear regression with multiply imputed data and control for baseline value of the outcome.  Column 1: Net intervention effect on year-2 outcome for attendees relative to all controls, adjusted for random assignment pair and community.  Column 2: Effect of any attendance compared to no attendance on year-2 outcome value within intervention arm, adjusted for community.  Column 3: Dose-response effect of number of activities attended on year-2 outcome value among attendees, adjusted for community. | | | | | | |
